# A novel cuproptosis-related lncRNA signature predicts the prognosis and immune landscape in bladder cancer

**DOI:** 10.3389/fimmu.2022.1027449

**Published:** 2022-11-14

**Authors:** Yuchen Bai, Qi Zhang, Feng Liu, Jing Quan

**Affiliations:** Urology and Nephrology Center, Department of Urology, Zhejiang Provincial People’s Hospital, Affiliated People’s Hospital, Hangzhou Medical College, Hangzhou, China

**Keywords:** cuproptosis, lncRNA, prognosis, immune, BLCA

## Abstract

**Background:**

Bladder cancer (BLCA) is one of the deadliest diseases, with over 550,000 new cases and 170,000 deaths globally every year. Cuproptosis is a copper-triggered programmed cell death and is associated with the prognosis and immune response of various cancers. Long non-coding RNA (lncRNA) could serve as a prognostic biomarker and is involved in the progression of BLCA.

**Methods:**

The gene expression profile of cuproptosis-related lncRNAs was analyzed by using data from The Cancer Genome Atlas. Cox regression analysis and least absolute shrinkage and selection operator analysis were performed to construct a cuproptosis-related lncRNA prognostic signature. The predictive performance of this signature was verified by ROC curves and a nomogram. We also explored the difference in immune-related activity, tumor mutation burden (TMB), tumor immune dysfunction and exclusion (TIDE), and drug sensitivity between the high- and low-risk groups.

**Results:**

We successfully constructed a cuproptosis-related lncRNA prognostic signature for BLCA including eight lncRNAs (RNF139-AS1, LINC00996, NR2F2-AS1, AL590428.1, SEC24B-AS1, AC006566.1, UBE2Q1-AS1, and AL021978.1). Multivariate Cox analysis suggested that age, clinical stage, and risk score were the independent risk factors for predicting prognosis of BLCA. Further analysis revealed that this signature not only had higher diagnostic efficiency compared to other clinical features but also had a good performance in predicting the 1-year, 3-year, and 5-year overall survival rate in BLCA. Notably, BLCA patients with a low risk score seemed to be associated with an inflamed tumor immune microenvironment and had a higher TMB level than those with a high risk score. In addition, patients with a high risk score had a higher TIDE score and a higher half maximal inhibitory concentration value of many therapeutic drugs than those with a low risk score.

**Conclusion:**

We identified a novel cuproptosis-related lncRNA signature that could predict the prognosis and immune landscape of BLCA.

## Introduction

Bladder cancer (BLCA) is one of the deadliest diseases, with over 550,000 new cases and 170,000 deaths globally every year (1, 2). Despite certain pathogenic factors for the development of BLCA, including advanced age and cigarette smoking, the precise mechanism was not clear yet (3). In the past 30 years, the treatment of BLCA has evolved from surgery to a multidisciplinary approach including surgery, chemoradiotherapy, and immunotherapy (4, 5). However, the prognosis of patients with muscle-invasive BLCA was less favorable, and the 5-year survival rate was less than 50% (6). Moreover, BLCA is prone to distant metastasis when it invaded the sarcolemma (7). Until now, there is no ideal prognostic marker or signature for predicting prognosis of BLCA.

Cuproptosis is a copper-triggered programmed cell death (8). Copper is a fundamental trace element in many biological processes. Excess copper contributed to the aggregation of lipoylated dihydrolipoamide S-acetyltransferase intracellularly, leading to proteotoxic stress and cuproptosis (9). Emerging lines of evidence suggested that copper level could affect the oncogenesis and progression of cancer (10). Moreover, cuproptosis-related signature could predict the prognosis and immune response in various types of cancers, including renal cell carcinoma (11), hepatocellular carcinoma (12), and melanoma (13).

Long non-coding RNAs (lncRNAs), a series of RNA molecules with a transcript length of more than 200 nt, could regulate gene expression *via* interacting with protein, RNA, and DNA (14). Accumulating studies reported that lncRNA could serve as prognostic biomarker and be involved in the progression of BLCA. An m6A-related lncRNA signature could predict prognosis and immune landscape in BLCA (15). Moreover, lncRNA LNMAT2 could promote lymphatic metastasis in BLCA (16). As far as we know, the prognostic value of cuproptosis-related lncRNAs and their correlation with immune landscape in BLCA had not been fully elucidated. In this study, we aimed to develop a novel cuproptosis-related lncRNA signature to predict the prognosis and immune landscape of BLCA.

## Materials and methods

### Data extraction and processing

**Figure 1:**
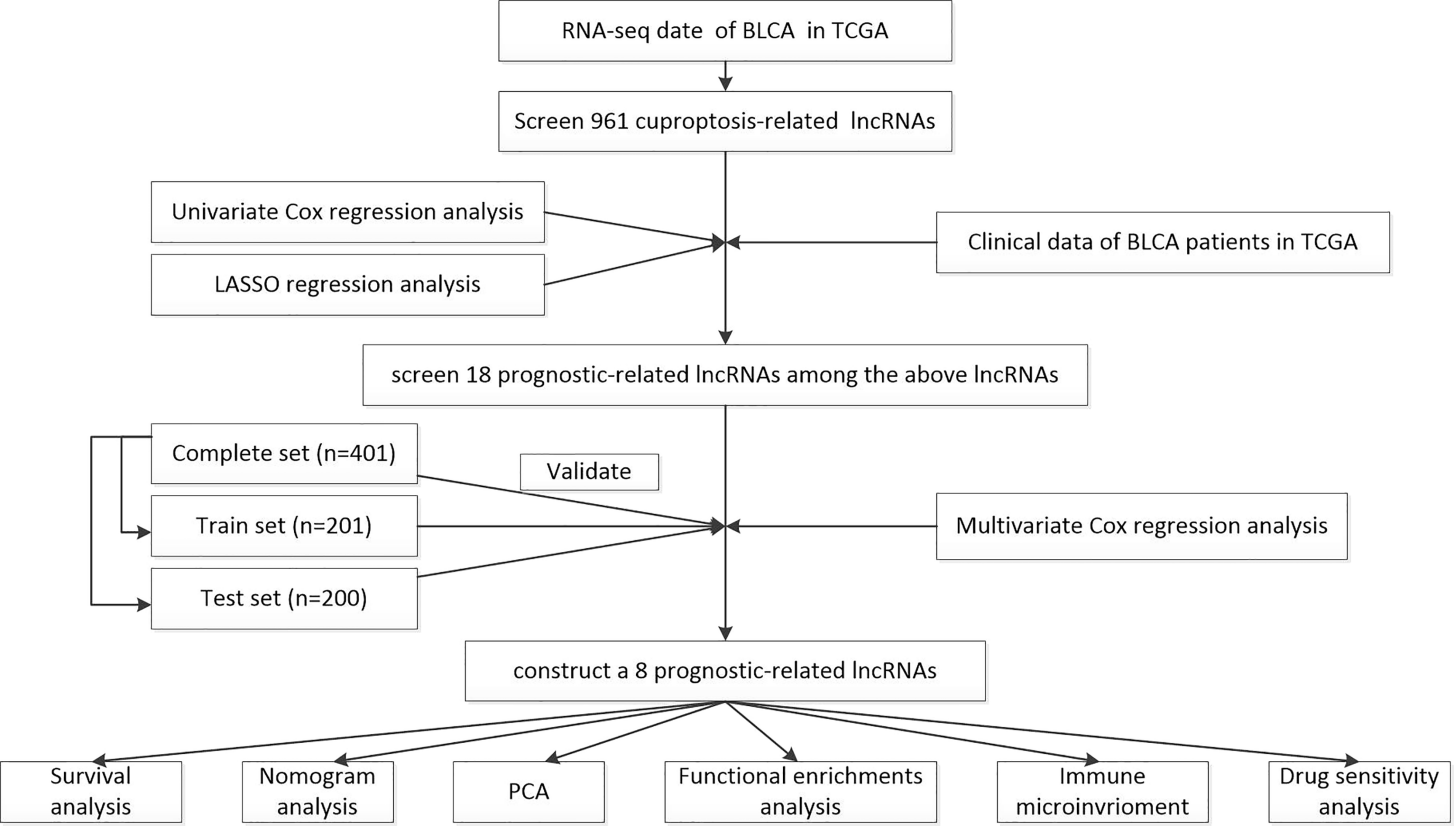
The workflow of the current study.

The workflow of the current study is shown in **Figure 1**. The transcriptomic data and clinical features of BLCA patients were downloaded from The Cancer Genome Atlas database (TCGA, https://portal.gdc.cancer.gov/), including 409 tumor samples and 19 normal samples. Strawberry Perl (version 5.30.0.1-64bit, https://strawberryperl.com/) was used to extract the useful information, such as the fragment per kilobase million format (FPKM) and the complete pathological information of each clinical sample. Then, Perl programming language was applied to distinguish mRNA and lncRNA. Moreover, simple nucleotide variation (SNV) data and masked somatic mutation data were also downloaded from the TCGA database, and used to calculate the mutational burden of BLCA. The cuproptosis-related genes were collected from the available online literatures, including NFE2L2, NLRP3, ATP7A, ATP7B, SLC31A1, FDX1, LIAS, LIPT1, LIPT2, DLD, DLAT, PDHA1, PDHB, MTF1, GLS, CDKN2A, DBT, GCSH, and DLST (17–21).

### Screening the differentially expressed cuproptosis-related lncRNAs

Limma package by R language (version 4.2.1, https://www.r-project.org/) was used to screen the differentially expressed genes. Then, Pearson’s correlation analysis was performed to assess the association between cuproptosis-related genes and cuproptosis-related lncRNAs. When Pearson’s correlation coefficient was greater than 0.4 and *p*-value was less than 0.001, these lncRNAs were considered to be related to cuproptosis and were statistically significant. The above result was visualized by using a Sankey diagram.

### Constructing a novel prognostic risk model of cuproptosis-related lncRNAs

By combining candidate lncRNAs with clinical data, the information about the expression and survival status of each lncRNA in clinical samples was obtained. Then, using the R package, the BLCA dataset downloaded from the TCGA database was randomly divided into two groups: train group and test group. The train group was used to construct the cuproptosis-related lncRNA signature, and the test group was used to validate the signature. Finally, the risk model was applied in the complete group. To further verify the prognosis-related lncRNAs among those candidate lncRNAs, univariate Cox regression analysis was performed (*p* < 0.05).

Next, least absolute shrinkage and selection operator (LASSO) regression analysis and lambda spectra are used to screen prognosis-related lncRNAs in order to prevent overfitting when constructing a prognostic risk model. Our research modified the LASSO regression analysis as follows: Run 1,000 cycles, and set 1,000 random stimuli in each cycle. In the next step, record the frequency of each pair of LASSO regression models that are repeated 1,000 times, and select the pairs with a frequency of more than 100 times to perform multivariate Cox proportional hazard regression analysis and build the model. Then, calculate the AUC values of these models. When the AUC value reaches the maximum value, it indicates that the model is the best candidate model. In this study, the risk score is obtained by the following formula: Risk Score= $\;\sum^{}\text{inCoef}\left(\text{i}\right)*\text{Expr}\left(\text{i}\right).\;$ In the above formula, inCoef(i) means the corresponding correlation coefficient of each lncRNA, while Expr(i) represents the corresponding standardized expression level of each lncRNA. After classifying BLCA samples in a low- or a high-risk subgroup in the training set, the test set, or the entire TCGA BLCA dataset, we then drew the overall survival (OS) and progression-free survival (PFS) of BLCA patients with the Kaplan–Meier (KM) method using the “survival” R package. The association between risk and clinical characteristics was analyzed with chi-square test. In order to analyze the accuracy of this risk model in the prognosis of BLCA, we also generated ROC curves and the consistency index (C-index) with “glmnet” and “timeROC” packages. Considering risk score and clinical characteristics, we then drew a nomogram and a calibration curve to evaluate the predictive power of a prognostic module in 1-, 3-, and 5-year OS using the “rms” package with the Hosmer–Lemeshow test.

### Principal components analysis and functional enrichments analysis

In order to visualize the expression patterns of cuproptosis-related lncRNAs in BLCA samples, principal components analysis (PCA) was conducted with the “scatterplot3d” package. After screening differential genes between the low- and high-risk groups, we performed Gene Ontology (GO) and KEGG pathways analysis with the “clusterProfiler” and “enrichplot” packages with FDR < 0.05 as threshold value. GO components included biological process (BP), cellular component (CC), and molecular function (MF).

### Immune infiltration

In order to evaluate the difference in immune infiltration between the high- and the low-risk group, we performed GSVA using the “reshape2” package and the “GSEABase” package. Furthermore, the methods such as XCELL (22), TIMER (23), QUANTISEQ (24), MCPCOUNTER (25), EPIC (26), CIBERSORT (27), and CIBERSORT-ABS (28) were applied to explore the relationship between risk scores and tumor-infiltrating immune cells. The Wilcoxon signed-rank test was used to analyze the differences in the content of immune infiltrated cells explored by the above methods between the high-risk and the low-risk group. Moreover, the relationship between risk scores and immune infiltrated cells was displayed by Spearman correlation analysis. A lollipop diagram was used to display the above results. The operation was performed using the ggplot2 package for R.

### Tumor mutation burden and drug sensitivity analysis

The somatic mutation data were also obtained from the TCGA website. The TMB oncoplot in the high-risk and low-risk groups was drawn with the “maftools” package. The KM method was used to draw the OS curve in the high and low TMB score group. After obtaining the tumor immune dysfunction and exclusion (TIDE) score from their website (http://tide.dfci.harvard.edu), we then analyzed the difference of TIDE score in the low- and high-risk groups of BLCA samples. Moreover, half maximal inhibitory concentration (IC_50_) values of different drugs in the high- and low-risk groups of BLCA samples were calculated with the “pRRophetic” R package.

## Results

### Defining the cuproptosis-related lncRNAs with prognostic significance

**Figure 2:**
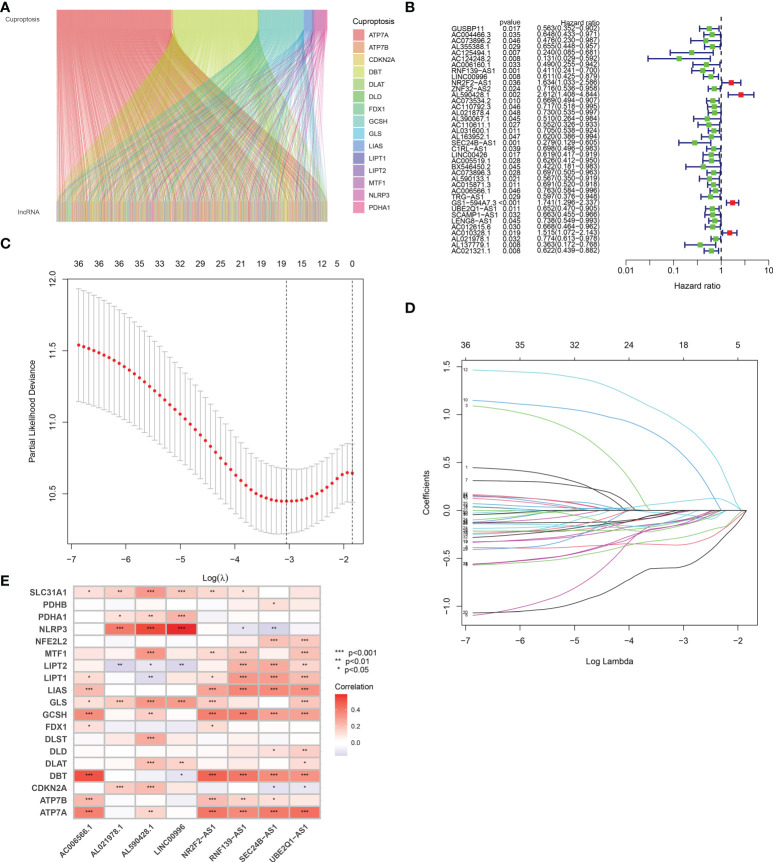
Defining the cuproptosis-related lncRNAs with prognostic significance in BLCA. **(A)** A total of 961 cuproptosis-related lncRNAs were identified in BLCA. **(B)** The forest plot revealed cuproptosis-related lncRNAs with significant prognostic value. **(C)** The 10-fold cross-validation of variable selection in the least absolute shrinkage and selection operator (LASSO) algorithm. **(D)** Correlation of lncRNAs with cuproptosis-related genes in prognostic signature. **(E)** The correlation between prognostic signature and cuproptosis-related genes. *p < 0.05, **p < 0.01, and ***p < 0.001.

As shown in **Figure 2A**, a total of 961 cuproptosis-related lncRNAs were identified in BLCA (|Pearson *R*| > 0.4 and *p* < 0.001). Based on these 961 lncRNAs, we performed univariate Cox analysis to identify these lncRNAs with prognostic significance. As a result, a total of 18 prognostic cuproptosis-related lncRNAs, namely, AC004466.3, AC125494.1, AC124248.2, RNF139-AS1, LINC00996, NR2F2-AS1, AL590428.1, AC073534.2, AL163952.1, SEC24B-AS1, LINC00426, BX546450.2, AL590133.1, AC006566.1, GS1-594A7.3, UBE2Q1-AS1, AL021978.1, and AL137779.1, were obtained (**Figure 2B**, *p* < 0.05).

### Development of a cuproptosis-related lncRNA prognostic signature

**Figure 3:**
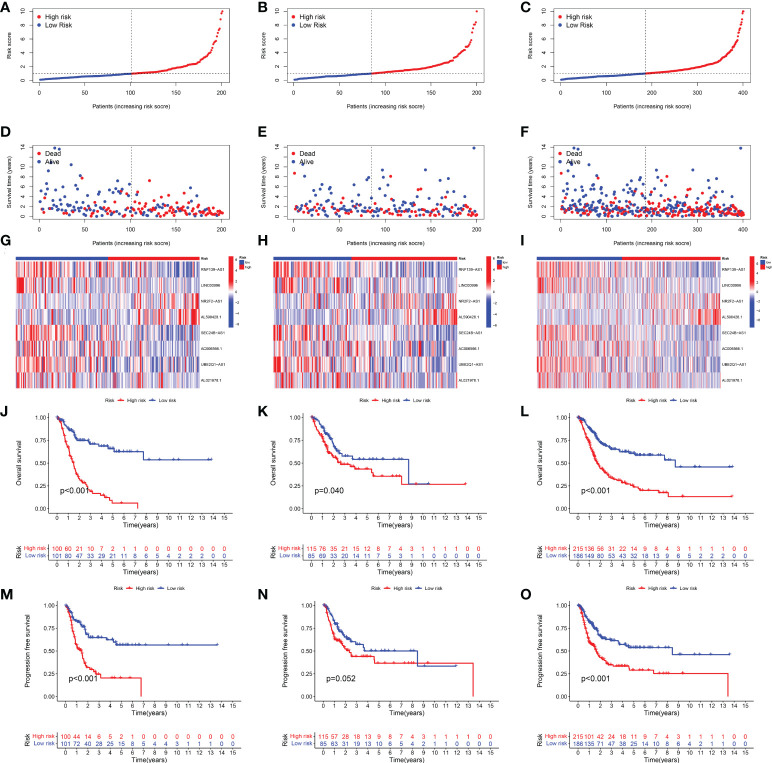
Development of the cuproptosis-related lncRNA prognostic signature in BLCA. The risk score **(A–C)**, survival status **(D–F)**, and gene expression **(G–I)** of prognostic signature in the training set, test set, and complete set. Kaplan–Meier survival curves of overall survival (OS, **J–L**) and progression-free survival (PFS, **M–O**) in the high- and low-risk groups of BLCA in the train set, test set, and complete set.

**Figure 4:**
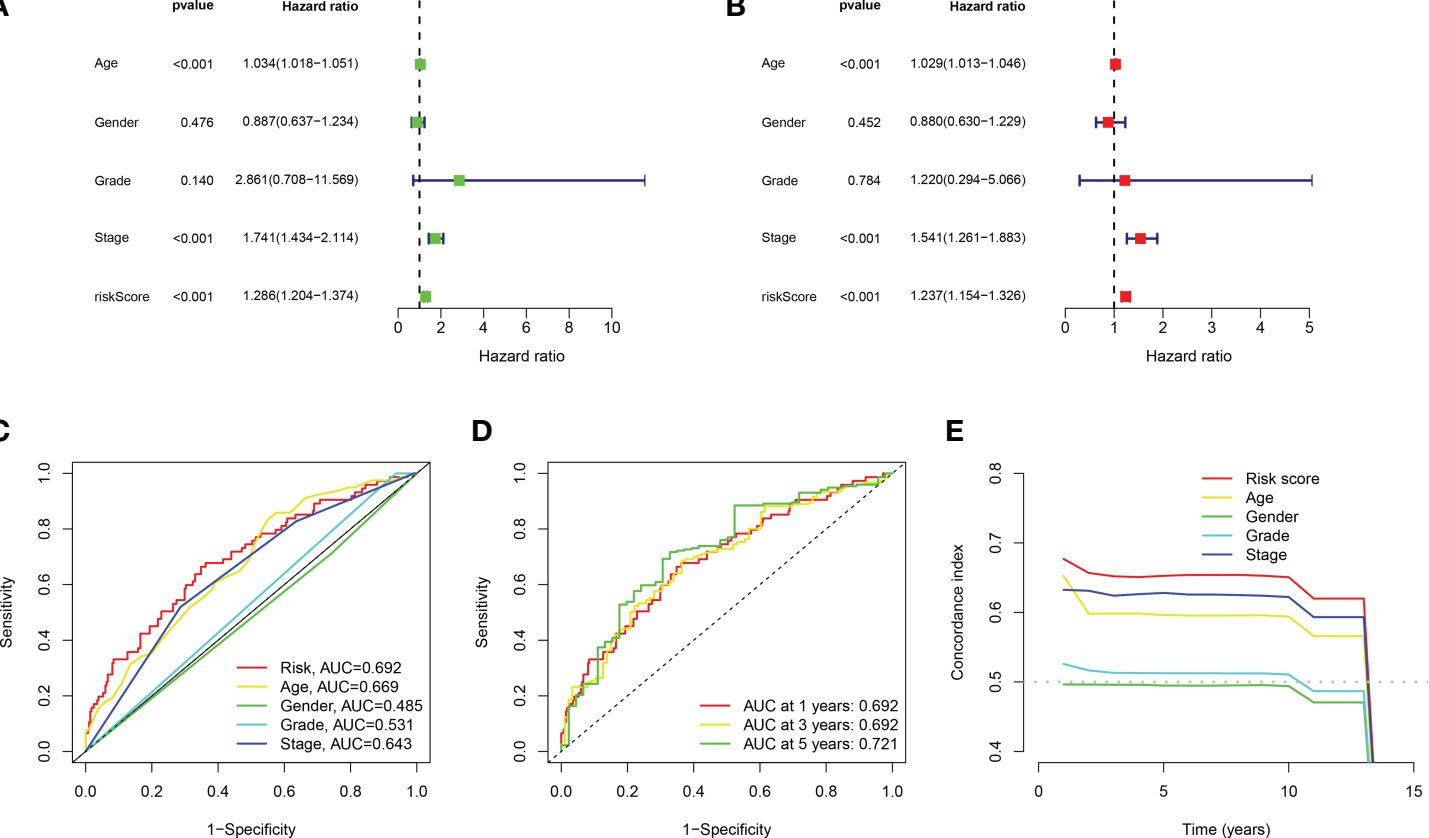
Evaluation about the accuracy of the cuproptosis-related lncRNA prognostic signature in BLCA. **(A, B)** Univariate Cox regression analysis considering risk score and clinical characteristics. **(C)** ROC curve considering risk score and clinical characteristics. **(D)** ROC curve of 1-year, 3-year, and 5-year overall survival. **(E)** C-index curve of the prognostic signature.

Based on the above 18 prognostic cuproptosis-related lncRNAs, we then performed a LASSO Cox regression analysis to construct a cuproptosis-related lncRNA prognostic signature in BLCA. As a result, this multi-Cox proportional risk model consisted of eight cuproptosis-related lncRNAs. **Figures 2C, D** revealed the coefficient and partial likelihood deviance of the multi-Cox proportional risk model. The correlation between cuproptosis-related genes and eight prognostic lncRNAs are shown in **Figure 2E**. The risk score of each BLCA case was calculated with the multivariate Cox regression formula: RNF139-AS1× (−0.478075619) + LINC00996× (−0.683046976) + NR2F2-AS1× (1.081783641) + AL590428.1× (1.423045357) + SEC24B-AS1× (−1.138326443) + AC006566.1× (−0.296157307) + UBE2Q1-AS1× (−0.360369461) + AL021978.1× (−0.26913395). TCGA BLCA cases were clustered in the high- and low-risk groups with a medium risk score as the cutoff value in the train set, the test set, and the complete set. The risk core, survival status, and gene expression of the train set, the test set, and the complete set are shown in **Figures 3A–I**. As expected, significant poor OS was obtained in BLCA cases with a high risk score in the train set (**Figure 3J**, *p* < 0.001), the test set (**Figure 3K**, *p* = 0.04), and the complete set (**Figure 3L**, *p* < 0.001). Unfortunately, although this model was a good predictor of PFS in BLCA patients in both train and complete groups (**Figures 3M, O**), it was not significant in the test group (**Figure 3N**). Further univariate and multivariate Cox analysis suggested that age, clinical stage, and risk score were the independent risk factors for prognosis of BLCA (**Figures 4A, B**).

### Evaluation of the predictive power of the cuproptosis-related lncRNA prognostic signature

**Figure 5:**
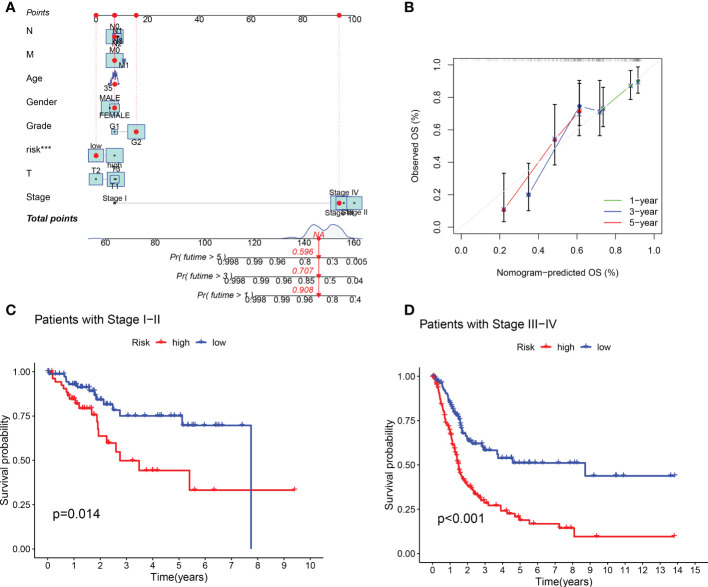
Construction of predictive nomogram and survival curve based on subgroup analysis. **(A, B)** A nomogram considering clinicopathological variables and risk scores predicts overall survival in BLCA. **(C, D)** Survival curve of the high-/low-risk group in different stages of BLCA patients.

Compared with the AUC of the ROC curve about age, sex, tumor grade, and clinical stage, the AUC of the ROC curve of risk score was the highest (**Figure 4C**). The AUC was 0.692, 0.692, and 0.721 for the 1-, 3-, and 5-year ROCs, respectively (**Figure 4D**). The 10-year C-index in the cuproptosis-related lncRNA prognostic signature was also higher than other clinical features (**Figure 4E**). These lines of evidence revealed that the cuproptosis-related lncRNA prognostic signature had a better predictive power compared to other clinicopathological characteristics. In order to predict the survival possibility of BLCA patients at 1, 3, and 5 years, we also constructed a nomogram considering clinical characteristics and risk scores, and the results are shown in **Figures 5A, B**. As expected, the calibration curves showed good agreement between the nomogram and the predicted results in 1-, 3-, and 5-year OS (**Figure 5B**). Moreover, we also verified this prognostic signature in different clinical stages. The result also suggested that BLCA patients with a high risk score had a poor OS in both stage I–II (**Figure 5C**, *p* = 0.014) and stage III–IV groups (**Figure 5D**, *p* < 0.001).

### The principal components analysis and biological pathways analyses

**Figure 6:**
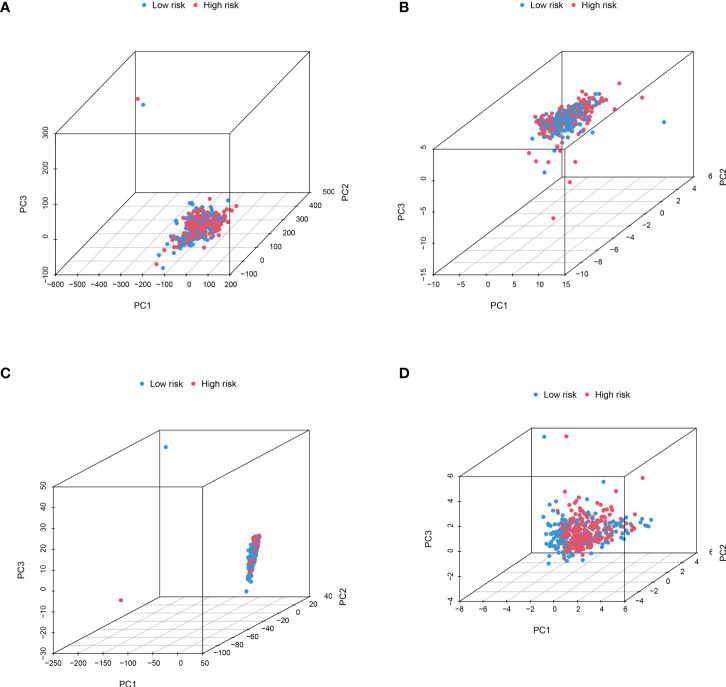
PCA considering the different gene profiles of BLCA patients. Comparing the gene module **(A)**, cuproptosis-related gene module **(B)**, cuproptosis-related lncRNA module **(C)**, and cuproptosis-related lncRNA prognostic module **(D)** could more clearly cluster BLCA cases into high- and low-risk groups.

**Figure 7:**
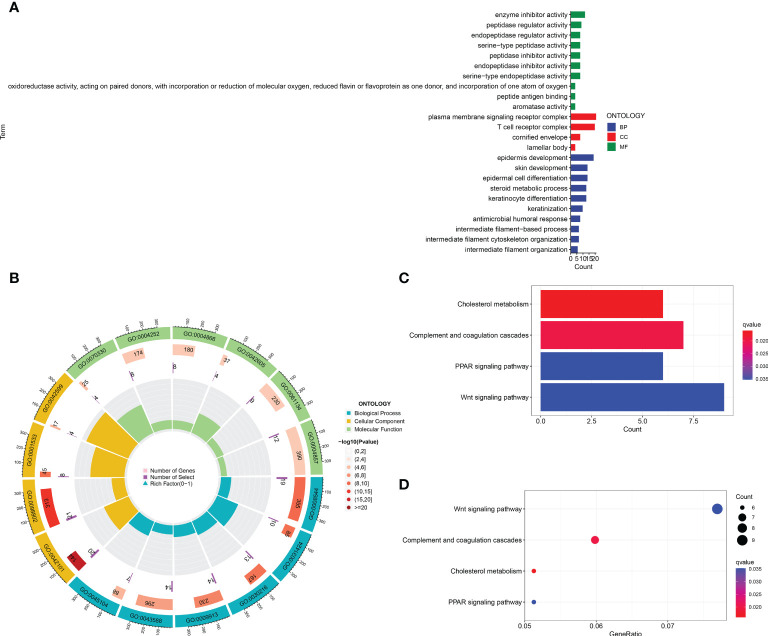
GO and KEGG analysis. The enrichment items in GO **(A, B)** and KEGG pathway **(C, D)** analysis.

In order to identify whether the cuproptosis-related lncRNA prognostic signature could cluster BLCA cases into the high- and low-risk groups, PCA was performed. Interestingly, comparing the gene module (**Figure 6A**), cuproptosis-related gene module (**Figure 6B**), cuproptosis-related lncRNA module (**Figure 6C**), and cuproptosis-related lncRNAs prognostic module (**Figure 6D**) could more clearly cluster BLCA cases into the high- and low-risk groups. In order to clarify the difference between the high- and low-risk groups, we also performed GO and KEGG pathway analysis using the differentially expressed genes between these two groups. GO analysis revealed that these cuproptosis-associated lncRNAs were mainly linked to epidermis development, omega-hydroxylase P450 pathway, T-cell receptor complex, aromatase activity, and enzyme inhibitor activity (**Figures 7A, B**). Moreover, KEGG pathway analysis suggested the involvement in cholesterol metabolism, complement and coagulation cascades, PPAR signaling pathway, and Wnt signaling pathway (**Figures 7C, D**).

### Immune infiltration

**Figure 8:**
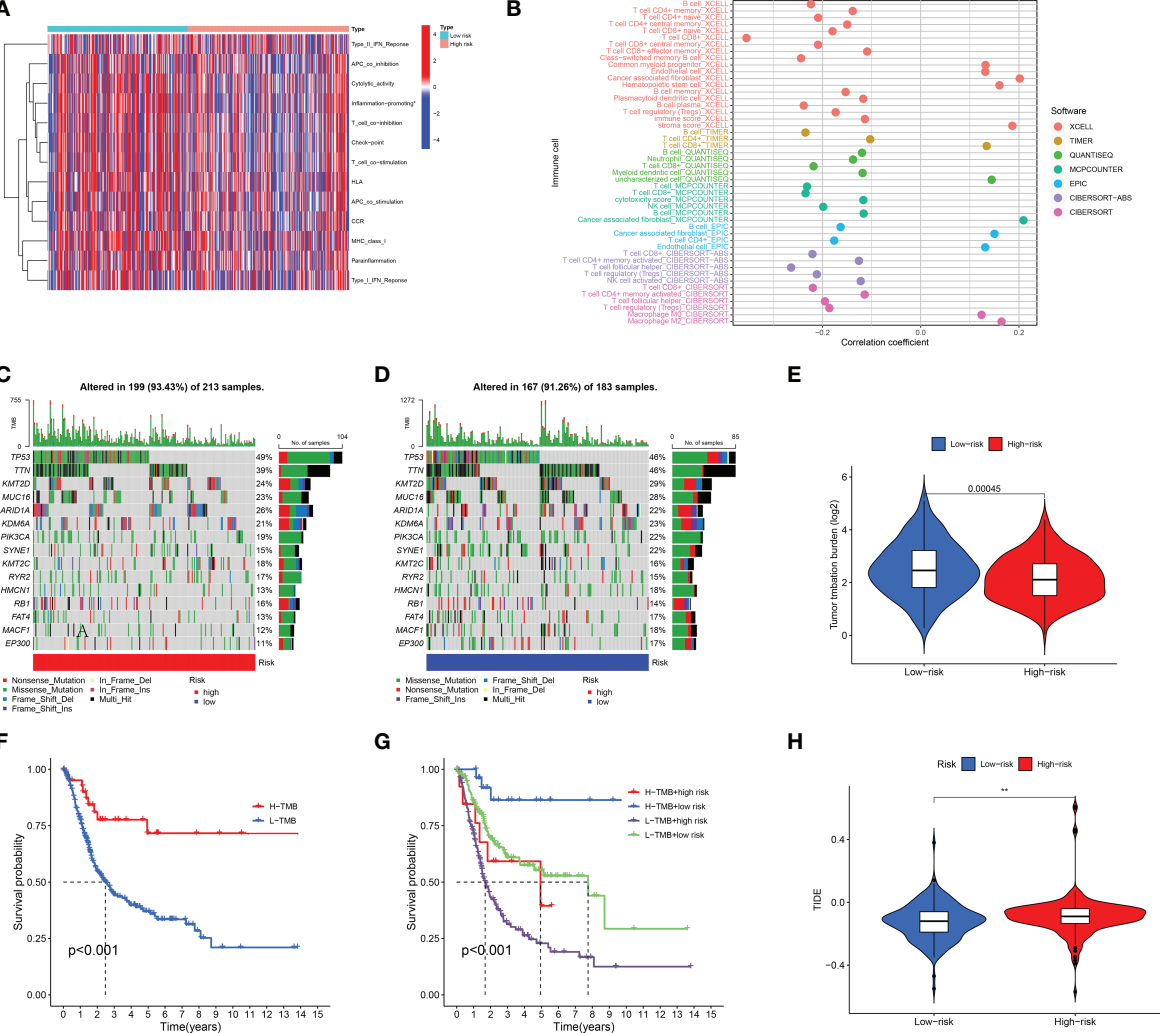
Immune-related activity, TMB, and TIDE analysis. The difference in immune-related activities **(A)** and tumor-infiltrating immune cells **(B)**. The difference in somatic mutation characteristics **(C, D)** between the high- and low-risk groups. **(E)** TMB between the low-risk and high-risk groups of BLCA patients. **(F, G)** Survival curve in the different groups of BLCA patients. **(H)** TIDE scores between the low-risk and high-risk groups of BLCA patients. **(F)** shows that among bladder cancer patients, the high tumor mutation burden group (H-TMB) was associated with a better prognosis. **p < 0.01.

We further surveyed the difference between the high- and low-risk groups in common immune-related activities. However, no significant difference was obtained between the high- and low-risk groups in common immune-related activities (**Figure 8A**). To explore the relationship between the immune system and the risk assessment model, recognized methods, including XCELL, TIMER, QUANTISEQ, MCPCOUNTER, EPIC, CIBERSORT-ABS, and CIBERSORT, were used. As a result, the lollipop chart (**Figure 8B**) shows that patients in the high-risk group are positively correlated with tumor-infiltrating immune cells (such as cancer-related fibroblasts, common myeloid progenitor cells, endothelial cells, hematopoietic stem cells, M0 macrophages, M2 macrophages, stroma score, and uncharacterized cells), and they are negatively correlated with memory B cells, plasma B cells, B cells, class-switched memory B cells, CD4+ central memory T cells, CD4+ memory activated T cells, CD4+ memory T cells, CD4+ naïve T cells, CD4+ T cells, follicular helper T cells, regulator T cells (Tregs), and T cells by Spearman correlation analysis (see **Supplement 1** for details).

### TMB, TIDE, and drug sensitivity

**Figure 9:**
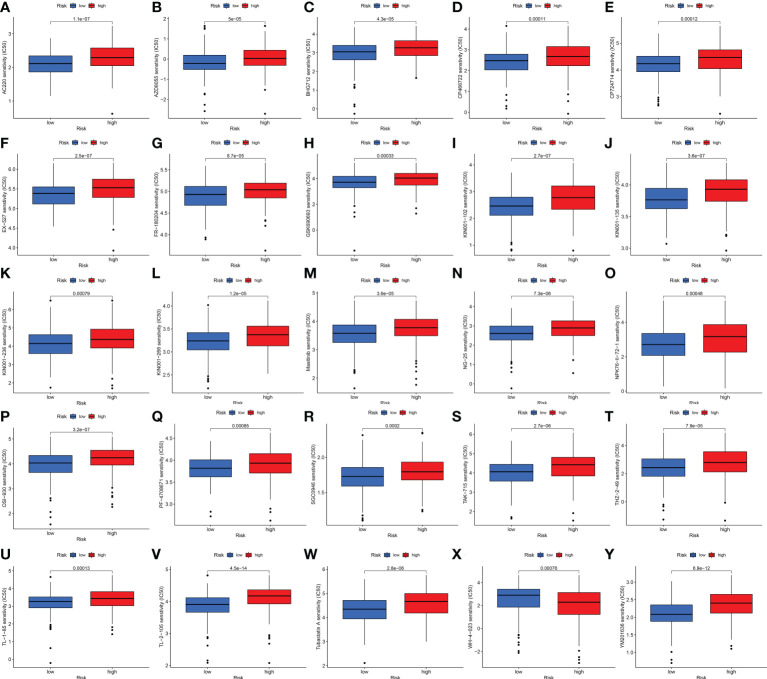
Drug sensitivity analysis. **(A–Y)** BLCA patients with a high risk score had a higher IC_50_ value of many therapeutic drugs compared with patients with a low risk score.

We also explored the difference between the high- and low-risk groups in TMB, TIDE, and drug sensitivity. For TMB analysis, we found that the 15 most highly mutated genes were TP53, TTN, KMT2D, MUC16, ARID1A, KDM6A, PIK3CA, SYNE1, KMT2C, RYR2, HMCN1, RB1, FAT4, MACF1, and EP300 in BLCA. As shown in **Figures 8C, D**, the mutation rate of these genes was higher in the high-risk group, including TP53, ARID1A, KMT2C, RYR2, and RB1. On the contrary, the mutation rate of the remaining genes was higher in the low-risk group, including TTN, KMT2D, MUC16, KDM6A, PIK3CA, SYNE1, HMCN1, FAT4, MACF1, and EP300. Interestingly, BLCA patients with a low risk score had a higher TMB level (**Figure 8E**, *p* = 0.00045). Further analysis suggested that BLCA patients with a low TMB and a high risk score had a poor OS rate (**Figures 8F, G**, *p* < 0.001). As shown in **Figure 8H**, BLCA patients with a high risk score had a higher TIDE score than those with a low risk score (*p* < 0.01). Further analysis also revealed that BLCA patients with a high risk score had a higher IC_50_ value of many therapeutic drugs than those with a low risk score (**Figures 9A–Y**). These results suggested that BLCA patients with a high risk score may be more likely to be resistant to chemotherapy and immunotherapy.

## Discussion

BLCA is the second most frequent genitourinary cancer, with a growing incidence worldwide (29). The symptoms of BLCA were similar to urinary infection, thus delaying timely diagnosis (30). It is estimated that 1/4 of the initially diagnosed cases was muscle-invasive BLCA (31). Moreover, BLCA is characterized by high recurrence rate and poor prognosis. The prognosis of patients with muscle-invasive BLCA was less favorable, and the 5-year survival rate was less than 50% (6). Thus, there is an urgent need to identify the reliably prognostic biomarkers and novel therapy targets of BLCA.

Metal ions are essential micronutrients in mammals. However, insufficient or excessive metal ions do harm to health and lead to cell death (8). A recent study performed by Tsvetkov et al. reported that excess copper contributes to the aggregation of lipoylated dihydrolipoamide S-acetyltransferase intracellularly, leading to a novel of mitochondrial cell death called “cuproptosis” (8, 17). Increasing lines of evidence had constructed a cuproptosis-related signature for the prognosis and immune response of certain types of cancer (32–34).

Accumulating studies have reported the significant role of lncRNAs in the development and progression of BLCA. lncRNA-RMRP could accelerate the proliferation, migration, and invasion of BLCA cells (35). By regulating E2F1, lncRNA-SLC16A1-AS1 could promote metabolic reprogramming in BLCA (36). Moreover, lncRNA TUC338 was considered as a diagnostic biomarker for BLCA (37). Another meta-analysis revealed that lncRNAs could serve as a diagnostic and prognostic biomarker in BLCA (38). However, the prognosis value of cuproptosis-related lncRNAs and their correlation with immune landscape in BLCA had not been fully clarified.

We first constructed a cuproptosis-related lncRNA prognostic signature for BLCA including eight lncRNAs (RNF139-AS1, LINC00996, NR2F2-AS1, AL590428.1, SEC24B-AS1, AC006566.1, UBE2Q1-AS1, and AL021978.1). Further analysis revealed that this prognostic signature not only had higher diagnostic efficiency compared to other clinical features but also had a good performance in predicting the 1-year, 3-year, and 5-year OS in BLCA. Actually, many studies had reported that cuproptosis-related lncRNA signature could serve as a prognostic biomarker in many types of cancer. Liu et al. constructed cuproptosis-related lncRNAs as biomarkers of prognosis and the immune microenvironment in squamous cell carcinoma (39). Another cuproptosis-related lncRNA signature could guide the prognosis and immune microenvironment in osteosarcoma (40). Yun also developed a cuproptosis-related prognostic signature in uterine corpus endometrial carcinoma (41). In colon adenocarcinoma, the cuproptosis-related lncRNA signature could provide insights into the accurate prediction of prognosis (32).

GO and KEGG pathway analysis revealed that these cuproptosis-associated lncRNAs were mainly linked to epidermis development, omega-hydroxylase P450 pathway, T-cell receptor complex, aromatase activity and enzyme inhibitor activity, cholesterol metabolism, complement and coagulation cascades, PPAR signaling pathway, and Wnt signaling pathway. Among these signaling pathways, PPAR signaling exerts pleiotropic functions in cancer (42). Moreover, PPARγ inhibition was involved in the regulation of cell cycle, and proliferation and motility of BLCA (43). Moreover, Wnt signaling pathway was also associated with tumor cell invasion and metastasis in BLCA (44). The tumor microenvironment of BLCA was also associated with Wnt signaling pathway (45). Thus, cuproptosis-associated lncRNAs might regulate biological processes in BLCA *via* these pathways. Further study should be performed to verify these results. As mentioned above, our novel cuproptosis-related lncRNA signatures also predict the relationship between tumor-infiltrating immune cells and BLCA. These lncRNAs also play an important role in the immunity of other diseases. For example, previous studies have shown that LNC00996 is overexpressed in lung adenocarcinoma and squamous cell carcinoma, and is closely related to the immune system (46). In addition, AL590428.1 was downregulated in human pterygium fibroblasts, which is closely related to cell cycle and apoptosis (47).

As is known, the TIDE algorithm was performed to assess the clinical response to immune checkpoint inhibitor (ICI) treatment. A higher TIDE score indicates a greater likelihood of immune escape, meaning that patients treated with ICI have a limited response and a shorter survival time (11). Thus, another vital finding of our study was that BLCA patients with a high risk score had a higher TIDE score compared with patients with a low risk score. For TMB analysis, we found the 15 most highly mutated genes in BLCA. Among them, the mutation rate of TP53 was higher in the high-risk group. As we know, the transcription factor p53 is a key tumor suppressor that is inactivated in almost all cancers due to point mutations in the TP53 gene or overexpression of its negative regulator. The p53 protein is known as the “cellular gatekeeper” because of its role in promoting DNA repair, cell cycle arrest, or apoptosis in response to DNA damage (48). Moreover, IC_50_ is an important index for evaluating drug efficacy or sample response to treatment, and cancer patients with a higher IC_50_ value had a greater likelihood of drug resistance (49). In our study, BLCA patients with a high risk score had a higher IC_50_ value of many therapeutic drugs compared with patients with a low risk score. The above drugs contain AC220, AZD8055, BHG712, CP466722, CP724714, EX-527, FR-180204, GSJ690693, KIN001-102, KIN001-135, KIN001-236, KIN001-266, Masitinib, NG-25, NPK76-II-72-1, OSI-930, PF-4708671, SGC0946, TAK-715, THZ-2-49, TL-1-85, TL-2-105, Tubastatin A, WH-4-023, and YM201636. Among these drugs, as a novel receptor tyrosine kinase inhibitor, Masitinib (also known as AB1010) has been shown to have inhibitory activity and increase apoptosis in bladder TCC cells, and is positively correlated with PDGFRα and c-Kit receptor expression levels (50). Moreover, as a special HDAC6 inhibitor, Tubastatin A (TST) can affect cell growth and promote structural modifications in cancer cells and parasites, which are potential anti-*Toxoplasma gondii* chemotherapeutics (51, 52). Urdician et al. found that TST could enhance temozolomide−induced apoptosis and change the malignant phenotype of glioblastoma cells (53). Thus, our results suggested that BLCA patients with a high risk score may be more likely to be resistant to chemotherapy and immunotherapy, which may be used to guide the treatment of patients with BLCA.

The current study had several limitations that should be acknowledged. First of all, the data in this study come from a single source. Due to the deviation and limitation of commercial microarray, we cannot get verification from the GEO and ICGC database. Secondly, we did not verify the above lncRNAs, including their expression and mechanism in BLCA. Finally, the immune cell bubble map shows the results of immune infiltration in multiple platforms, which can be considered external validation in a sense and lacks rigor. Therefore, in the future, we should not only verify the accuracy and practicality of the above lncRNA signature by collecting a large number of clinical data, but also further explore the mechanism of these lncRNAs in BLCA.

## Conclusion

In conclusion, this novel cuproptosis-related lncRNA signature helps us not only to predict the prognosis of BLCA, but also to understand the immune landscape and drug sensitivity of BLCA. Further investigation is still needed to validate these findings.

## Data availability statement

Publicly available datasets were analyzed in this study. This data can be found here: the TCGA-BLCA (https://portal.gdc.cancer.gov/).

## Author contributions

JQ and YB conducted the formal analysis and wrote the original draft. FL and QZ performed project administration. JQ provided software and conducted data curation. YB, FL and QZ contributed to writing, reviewing, and editing the article. All authors contributed to the article and approved the submitted version.

## Funding

This study was supported by the General Project Funds from the Health Department of Zhejiang Province (Grant no. 2021439557), General Project Funds from the Health Department of Zhejiang Province (Grant no. 2022496245), and Project Funds of Zhejiang Provincial Science and Technology Department (Grant no. LY18H160040).

## Conflict of interest

The authors declare that the research was conducted in the absence of any commercial or financial relationships that could be construed as a potential conflict of interest.

## Publisher’s note

All claims expressed in this article are solely those of the authors and do not necessarily represent those of their affiliated organizations, or those of the publisher, the editors and the reviewers. Any product that may be evaluated in this article, or claim that may be made by its manufacturer, is not guaranteed or endorsed by the publisher.
